# Hunting-training dogs and companion dogs in the Netherlands are frequently exposed to highly pathogenic avian influenza (HPAI H5) and human H1N1 virus, 2021–2023

**DOI:** 10.1016/j.onehlt.2025.101134

**Published:** 2025-07-14

**Authors:** Mirjam B.H.M. Duijvestijn, Nancy N.M.P. Schuurman, Johannes C.M. Vernooij, Marian J. Broekhuizen, Erwin de Bruin, Benine S. Carrière, Judith M.A. van den Brand, Jaap A. Wagenaar, Frank J.M. van Kuppeveld, Herman F. Egberink, Cornelis A.M. de Haan, Josanne H. Verhagen

**Affiliations:** a Division of Infectious Diseases and Immunology, Department of Biomolecular Health Sciences, Faculty of Veterinary Medicine, Utrecht University, Utrecht, the Netherlands; b Division of Farm Animal Health, Department of Population Health Sciences, Faculty of Veterinary Medicine, Utrecht University, Utrecht, the Netherlands; c Division of Pathology, Department of Biomolecular Health Sciences, Faculty of Veterinary Medicine, Utrecht University, Utrecht, the Netherlands

**Keywords:** Serosurveillance, Zoonosis, Pandemic preparedness, Avian influenza, Canine, Antibodies, Europe

## Abstract

Dogs are susceptible to the currently circulating highly pathogenic avian influenza (HPAI) H5 and human H1N1_pdm2009_ (pandemic H1N1) viruses, yet little is known about the extent to which dogs are exposed to both these viruses. Here we investigated HPAI H5 and human H1N1_pdm2009_ virus exposure in domestic dogs–including dogs that participated in hunting-training–and investigated lifestyle factors associated with HPAI H5 virus exposure. We screened sera from 538 dogs, sampled between 2021 and 2023, for influenza A virus antibodies, using ELISA and hemagglutination inhibition assays (HAIs). We analyzed lung tissue and (naso)pharyngeal swabs for influenza A viruses using RT-qPCR. Seropositivity to HPAI H5 virus was more frequent (13.3 %) in hunting-training dogs than in companion dogs with unknown bird contact (3.7 %). In contrast, seropositivity to H1N1_pdm2009_ was more frequent in companion dogs (7.1 %) than in hunting-training dogs (0.7 %). Based on owner questionnaires, seropositivity to HPAI H5 by ELISA in hunting-training dogs was significantly associated with recent bird contact in/near water (odds ratio 6.9). No influenza A viruses were detected in 207 necropsy dogs and 180 (hunting) dogs. Our findings suggest that dogs are frequently exposed to zoonotic influenza A viruses, and we recommend dog owners to avoid dog contact with sick/dead birds.

## Highlights

• High seropositivity in dogs to highly pathogenic avian influenza (HPAI) H5 virus in the Netherlands during HPAI H5 endemicity in wild birds.
• HPAI H5-seropositivity higher in hunting-training dogs than companion dogs.
• HPAI H5-seropositivity in hunting-training dogs associated with recent bird contact in/near water.
• High seropositivity in dogs to human H1N1_pdm2009_ influenza A virus (IAV).
• H1N1_pdm2009_-seropositivity higher in companion dogs than hunting-training dogs.

## 1 Introduction

Since 2020, highly pathogenic avian influenza (HPAI) H5 viruses of the 2.3.4.4b subclade cause mass mortality in wild and domestic birds worldwide and have spilled over to more than 58 mammalian species [1] including domestic dogs [[2], [3], [4], [5], [6], [7], [8]]. Dogs naturally or experimentally (intranasal, epi-ocular or intra-tracheal) infected with HPAI H5 virus were asymptomatic or showed respiratory signs and death [2,4,5,[7], [8], [9], [10], [11], [12], [13]].

In addition to HPAI H5, dogs are susceptible to infection with other avian or mammalian influenza A viruses (IAV). Most remarkably, avian H3N2 and equine H3N8 IAVs caused respiratory disease outbreaks in kennels and shelters in North America and Asia [[14], [15], [16], [17]].

Human-origin H1N1_pdm2009_ IAV can naturally and experimentally infect dogs [6,[18], [19], [20]], and high (236/960) seropositivity has been reported in Chinese dogs [20]. Experimental, but not natural transmission of HPAI H5 or H1N1_pdm2009_ among dogs has been described [7,12,19]. While antibodies specific to HPAI H5 and H1 have been detected simultaneous in dogs [6], reassortment between these IAVs has not been detected. However, reassortant IAVs between H1N1_pdm2009_ and H3N2 IAVs [21,22], or H3N2 and H5N6 IAVs [23] have been isolated from the respiratory tract of dogs. Hence, dogs may act as “mixing vessels” for human and animal origin IAVs, resulting in novel IAVs.

Given the variation in signs and short period of IAV detection in dogs, antibody detection has been used to investigate HPAI H5 or H1N1_pdm2009_ exposure [6,[18], [19], [20]]. For instance, HPAI H5 specific antibodies were detected in all five dogs on a HPAI H5 infected poultry farm in Italy (2023), and in four of 194 hunting dogs in the United States (2023) [3,24]. Furthermore, antibodies to H1N1_pdm2009_ were detected in 13 of 882 and 236 of 960 dogs in China (2012−2013), in seven of 964 dogs in Italy (2009), and in eight of 496 dogs in Poland (2016–2017) [18,20,25,26]. Interestingly, 19 of 154 dogs sampled in the Netherlands (2019) had antibodies to H1N1_pdm2009_, two of 154 to HPAI H5 virus, and one dog was seropositive to both viruses [6]. Therefore, dogs can be exposed to HPAI H5 and H1N1_pdm2009_, yet the extent to which this occurs needs further exploration.

The primary aim of this study was to investigate exposure to HPAI H5 and H1N1_pdm2009_ IAV in domestic dogs, during HPAI H5 endemicity in wild birds in the Netherlands (2021−2023) [27]. Hunting-training dogs were included given their exposure to aquatic birds, as this may be a prerequisite for HPAI H5 exposure [[2], [3], [4], [5]]. As a secondary aim, we investigated lifestyle factors of hunting-training dogs in relation to HPAI H5 serostatus.

## 2 Methods

### 2.1 Study population

We explored HPAI H5 and H1 virus exposure in four different dog cohorts (Table 1).

**Table 1:** General description of four dog cohorts, the Netherlands, 2021–2023.

	Hunting-training	Bird-exposed	Companion	Necropsy
n	143	45	350	207
Sampling period	8/2022–6/2023	1/2022–10/2023	9/2022–2/2023	7/2021–11/2022
Mean age (median; IQR)	3.5 (3; 2–5)	4.1 (3; 1–7)	7.3 (8; 4–11)	3.9 (2; 1–7)^a^
Male/female ratio (male/female)	0.88 (67/76)	0.80 (20/25)	1.1 (182/161)^b^	0.95 (100/105)^b^
Hunting breed	143	19	63	30
Non-hunting breed	0	26	287	177

IQR: interquartile range.

a Age was unavailable for 14 dogs from the necropsy cohort.

b Sex was unavailable for seven dogs from the companion dog cohort and two from the necropsy dog cohort.

The hunting-training dog cohort (*n* = 143) consisted of retriever dogs that participated in hunting-training, hunting-trials or hunting of small game and had regular bird and/or water-contact in the past 6 months. Upon owner consent, sera, swabs, and questionnaires (Supplementary datafile S1) were conveniently collected at hunting-training or hunting-trials on 11 sampling days. Out of 143 dogs, one dog displayed clinical signs upon contact with a sick/dead bird.

The bird-exposed dog cohort (*n* = 45) consisted of dogs that had at least one dog-bird contact in the past six months. Upon owner consent, sera, swabs and questionnaires (Supplementary datafile S1) were conveniently collected by 26 veterinary practices. Out of 37 dogs, 11 displayed clinical signs upon contact with a sick/dead bird.

The companion dog cohort (*n* = 350) consisted of surplus sera sampled for diagnostic purposes (other than IAV infection).

The necropsy dog cohort consisted of surplus swabs and lung tissue from deceased dogs (*n* = 207) submitted for autopsy. Of these, 26 dogs displayed neurologic or respiratory inflammatory abnormalities.

### 2.2 Sample collection

#### 2.2.1 Ethical statement

Ethical approval for the collection of hunting-training or bird-exposed dog sera was granted by the Netherlands Central Authority for Scientific Procedures on Animals (CCD) under no.AVD10800202215874.

#### 2.2.2 Blood samples

Blood samples were collected from the cephalic vein in 5 ml serum tubes, centrifuged and serum stored at −20 °C until processing.

#### 2.2.3 (Naso)pharyngeal swabs or tissue samples

From 143 hunting-training dogs and 37 of 45 bird-exposed dogs, nasopharyngeal swabs were collected in RNA-shield medium (Zymo research, Irvine, US) and stored at −80 °C until processing. Additionally, from 207 necropsy dogs, pharyngeal swabs and lung tissue were collected and stored at −80 °C.

#### 2.2.4 Sample metadata

Data on age, sex, breed, sampling year and geographic location were available for all cohorts. From hunting-training and bird-exposed dogs, owner questionnaires provided information on the dog's lifestyle, health status and bird contact (not available for companion dogs and necropsy dogs).

### 2.3 Laboratory methods

#### 2.3.1 Antibody detection – ELISA

Antibodies binding to IAVs were detected in diluted dog sera (1:100) using in-house ELISAs coated with recombinant soluble trimeric hemagglutinin protein (HA; complete ectodomain) [6,28]. For detection of HPAI H5 clade 2.3.4.4c binding antibodies, the HA of A/Chicken/NL/14015526/2014 (H5N8, referred to as HPAI H5c) was used. For detection of H1 binding antibodies the HA of A/California/04/2009 (H1N1, referred to as H1) was used [6,28].

Subsequently, HPAI H5 clade 2.3.4.4c and/or H1 ELISA positive sera were investigated for binding to the HA of [29] HPAI H5 clade 2.3.4.4b virus (A/Common Tern/NL/26/2022 [H5N1]; referred to as HPAI H5b) and low pathogenic avian influenza (LPAI) H5 virus (A/Common Teal/NL/4/2022 [H5N2], referred to as LPAI H5) [28]. The genes for these HA proteins were expressed in HEK293ΔSia cells lacking all terminal Sia due to knockout of ST3Gal1/2/3/4/5/6 and ST6Gal1/2 [30].

The ELISA cutoff was set at 5 times the optical density (OD) value of a specific pathogen free dog serum [6]. Results were expressed as OD ratios (sample OD value/ cutoff; OD ratio ≥ 1 positive). A subset of 34 companion dog sera that tested negative to HPAI H5c and H1 was used as ELISA negative control cohort in the HPAI H5b and LPAI H5 ELISAs, and these sera reacted negative.

#### 2.3.2 Antibody detection – hemagglutination inhibition assay

The presence of IAV antibodies in ELISA positive sera and in the ELISA negative control cohort was further analyzed using hemagglutination inhibition assays (HAIs). The HAIs used SpyCatcher-mi3 virus-like nanoparticles covered with the HPAI H5c, LPAI H5 and H1 HA proteins used in the ELISAs [6,28]. Non-specific agglutinins were removed by pre-incubating the sera with 50 % packed chicken red blood cells for 1 h at 4 °C. The HAI antibody titer was defined as the highest dilution of the serum that showed complete inhibition of hemagglutination using 4–8 hemagglutinating units per 25 μl. The HAI cutoff was set to 1:40 [28]. None of the ELISA negative control cohort sera reacted positive in the HAIs.

The HPAI H5c-HAI displayed higher sensitivity than the HPAI H5b-HAI, based on HPAI H5c and HPAI H5b reference ferret antisera [28] and a selection of HPAI H5c ELISA positive dog sera (data not shown). As the amino acids that differed between HPAI H5c and HPAI H5b are not known to be important for changes in antigenicity [28], we only performed the HPAI H5c-HAI.

#### 2.3.3 Influenza A virus detection

To detect IAV RNA, a reverse transcriptase quantitative PCR (RT-qPCR) was used, targeting the matrix gene [28]. To optimize resources, pharyngeal swabs and lung tissue were pooled per three dogs (necropsy dogs) and (naso)pharyngeal swabs pooled per six dogs.

### 2.4 Data analysis

For data analysis and visualization, SPSS (SPSS Statistics for Windows, Version 28.0, IBM Corp. Armonk, US), GraphPad (GraphPadPrism version 10 for Windows, GraphPad Software, Boston, USA) and Datawrapper (Datawrapper GmbH, https://www.datawrapper.de) were used. Seroprevalence and confidence intervals were calculated using the Exact Binominal test. Seroprevalences were compared among cohorts using the two-proportions *Z*-test with Bonferroni correction for multiple comparisons. The ELISA mean rank OD ratios were compared using the Friedman test with Dunn's correction for multiple comparisons. To explore putative factors associated with HPAI H5c (hunting-training and companion dogs) or H1 (companion dogs) ELISA seropositivity, a univariable analysis was performed using the χ2 or Fisher's exact test (expected count <5) and the odds ratio (OR) with 95 % confidence interval (CI) was calculated. To calculate the OR in categories with zero events, 0.5 was added to all frequencies involved (Firth's correction) [31]. Factors with an OR ∼ 2 or higher or ∼ 0.5 or lower in the univariable analysis, were included in an automated (Forward and Backward) multivariable binary logistic regression model. Factors that contained categories with 0 values were excluded. The statistical significance level was set to *p* < 0.05 [28]. The bird-exposed dog cohort was excluded in these analyses due to the low sample size.

## 3 Results

### 3.1 Antibody detection

**Fig. 1:**
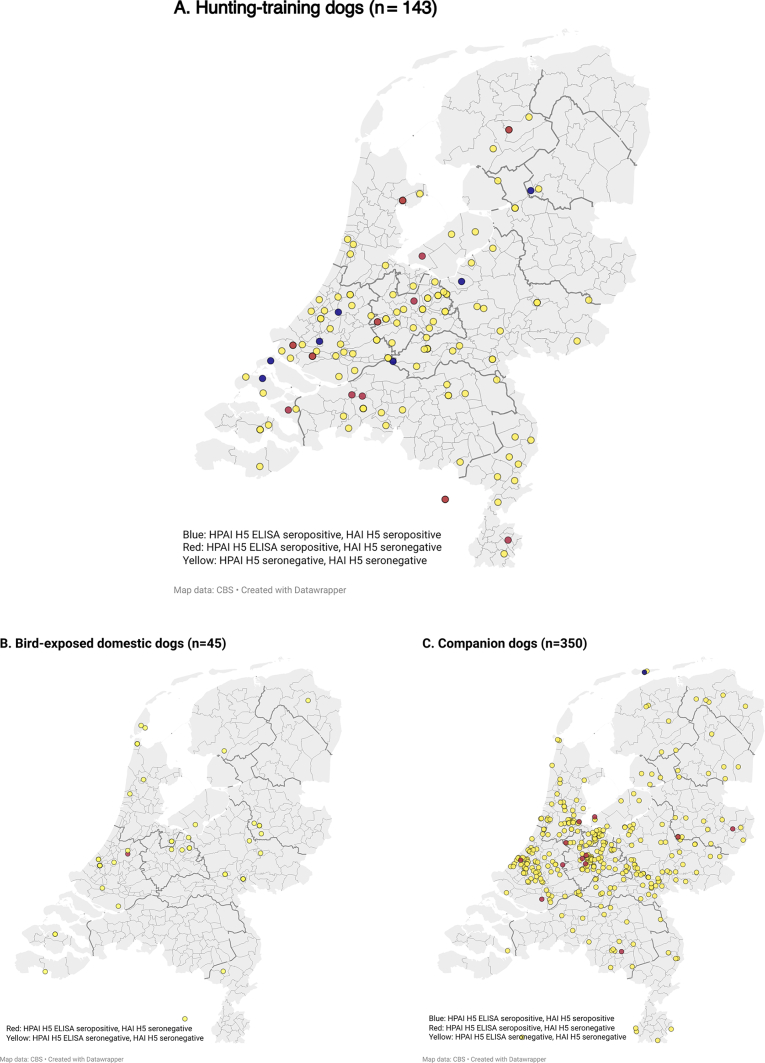
Spatial distribution of hunting-training dogs (*n* = 143), bird-exposed dogs (*n* = 45) and companion dogs (*n* = 350) serologically tested for HPAI H5 antibodies in the Netherlands, 2022-2023^a^. ELISA: enzyme linked immunosorbent assay; H: hemagglutinin; HAI: hemagglutination inhibition assay; HPAI: highly pathogenic avian influenza. The circles represent home addresses of dog owners. Locations were estimated using coordinates extracted from the postal code (companion dogs) or the municipality center (hunting-training dogs). ^a^Information on spatial location was available for 142 hunting-training dogs and 334 companion dogs.

The spatial distribution of HPAI H5c ELISA and/or HAI reactive hunting-training dogs, bird-exposed dogs and companion dogs is depicted in Fig. 1A,B,C.

#### 3.1.1 Hunting-training dogs

Based on ELISA, 20 of 143 hunting-training dog sera were positive for HPAI H5c and/or H1 antibodies (Table 2). Seropositivity to HPAI H5c was detected in 19 (13.3 %; 95 % CI:8.2–20.0) dogs while one serum was borderline reactive (OD ratio 0.88). One (0.70 %; 95 % CI:0.02–3.8) serum was seropositive to both HPAI H5c and H1.

**Table 2:** Seroprevalence of HPAI H5 clade 2.3.4.4c and human H1 in three dog cohorts, the Netherlands, 2022–2023.

		Hunting-training	Bird-exposed	Companion
n		143	45	350
Sampling period		8/2022–6/2023	1/2022–10/2023	9/2022–2/2023
HPAI H5c ELISA	Positive	19	1	13
	Percentage (95 % CI)	13.3 (8.2–20.0)	2.2 (0.06–11.8)	3.7 (2.0–6.3)
H1 ELISA	Positive	1	2	25
	Percentage (95 % CI)	0.70 (0.02–3.8)	4.4 (0.50–15.2)	7.1 (4.7–10.4)

ELISA: enzyme linked immunosorbent assay; H: hemagglutinin; HPAI: highly pathogenic avian influenza. CI: confidence interval.

**Fig. 2:**
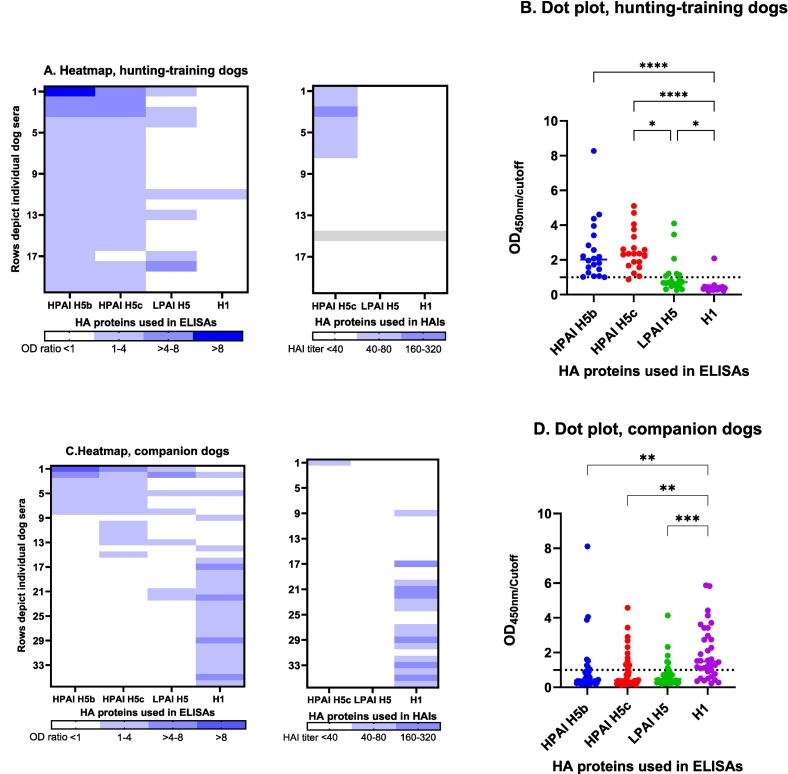
Heat maps and dot plots presenting serum samples from hunting-training dogs (A,B) and companion dogs (C,D) seropositive in ELISA to HPAI H5 clade 2.3.4.4 virus and/or LPAI H5 virus and/or H1N1_pdm2009_ virus, the Netherlands, 2022–2023. ELISA: enzyme linked immunosorbent assay; HAI: hemagglutination inhibition; HA: hemagglutinin; HPAI: highly pathogenic avian influenza; LPAI: low pathogenic avian influenza; OD: optical density measured at 450 nm; ****: *p* value <0.0001; *** p value<0.001; ** p value <0.01. Panel A and C: The level of ELISA or HAI binding activity of the sera was based on the OD ratio or HAI titer (white < cutoff). One dog serum could not be analyzed in the HAIs due to insufficient volume (grey in Panel A). The samples are sorted from highest to lowest OD ratio, from left to right. The dotted line (Panel B and D) represents the cut-off. The horizontal lines in the dot plots depict the median.

Based on ELISA, the sera that tested positive and the borderline reactive serum (n = 20) were tested for antibodies binding to a more recent HPAI H5b and to LPAI H5 HA. All 20 sera had binding antibodies to HPAI H5b. The mean rank HPAI H5c and HPAI H5b OD ratios were not significantly different (Fig. 2A, B). Seven of 20 sera were reactive to LPAI H5, but five of these seven sera were equal or higher reactive to HPAI H5c (Fig. 2A, B). The mean rank OD ratio was significantly higher for HPAI H5c than for LPAI H5 (Fig. 2B).

In HPAI H5-HAI, seven of 19 (36.8 %) HPAI H5c ELISA positive sera, were positive. None of the LPAI H5 ELISA reactive sera were positive in LPAI H5-HAI. The H1 ELISA positive serum was not positive in H1-HAI.

#### 3.1.2 Bird-exposed dogs

For bird-exposed dogs, one of 45 sera reacted to HPAI H5c, HPAI H5b and LPAI H5 in ELISA (Table 2) but not in HAI. In addition, two sera tested H1 ELISA positive, and were positive in H1-HAI.

#### 3.1.3 Companion dog cohort

Based on ELISA, 13 of 350 companion dog sera (3.7 %; 95 % CI: 2.0–6.3) tested HPAI H5c seropositive, while 25 of 350 sera (7.1 %; 95 % CI: 4.7–10.4) tested H1 seropositive (Fig. 2C, Table 2). Two sera reacted positive to both HPAI H5c and H1 but were negative in HAIs.

In the ELISA, the companion dog sera that tested positive in HPAI H5c and/or H1 (*n* = 36) were analyzed for antibodies binding to HPAI H5b and LPAI H5. Eight of 13 HPAI H5c seropositive samples reacted to HPAI H5b and the mean rank HPAI H5c and HPAI H5b OD ratios were not significantly different (Fig. 2D). Seven of 13 HPAI H5c positive sera reacted to LPAI H5, but five of these seven sera were equal or higher reactive to HPAI H5c than to LPAI H5 (Fig. 2C).

One of 13 HPAI H5c ELISA positive sera was positive in HPAI H5-HAI (Fig. 2D). None of seven LPAI H5 ELISA reactive sera were positive in LPAI H5-HAI, yet one of these sera was HPAI H5-HAI positive, and two sera were H1-HAI positive. Of 25 H1 ELISA positive companion dog sera, 16 (64.0 %) were H1-HAI positive (Fig. 2C).

#### 3.1.4 Comparison of ELISA reactivity among dog cohorts

The HPAI H5c ELISA seroprevalence of hunting-training dogs was significantly higher than of companion dogs (*p* = 0.001). The H1 ELISA seroprevalence of hunting-training dogs was significantly lower than of companion dogs (*p* < 0.001).

### 3.2 Influenza A virus detection

No IAV was detected in nasopharyngeal swabs of 143 hunting-training dogs, 37 bird-exposed dogs or pharyngeal swabs and lung tissue of 207 necropsy dogs.

### 3.3 Influenza A virus ELISA serostatus versus dog- or dog's lifestyle factors

#### 3.3.1 Hunting-training dogs

In hunting-training dogs, HPAI H5c seropositivity was more likely in dogs aged 3–6 years compared to younger dogs (OR = 2.0) (Table 3). Dogs that swim at least once per week (OR = 4.5) or walked at least once per week in an area with aquatic birds (OR = 6.9) were more likely HPAI H5c seropositive than dogs that did not. When the most recent bird contact within a 6 month time period was considered, HPAI H5c seropositivity was more likely in dogs that had been in contact with a bird in/near water (OR = 7.0), had been in contact with a suspected HPAI H5-infected bird (OR = 2.4) or had live-bird-in-beak contact (OR = 1.9). Remarkably, dogs used for hunting were less likely HPAI H5 seropositive than dogs used for training only (OR = 0.56).

**Table 3:** Analysis of lifestyle and dog-bird-contact factors and HPAI H5 clade 2.3.4.4 ELISA seropositivity in hunting-training dogs.

Factors	Categories	Total^a^		HPAI H**5** seropositive		OR (95 % CI)	*p*-Value
		n	%	n	%		
Age	0 < 3 year	63	44.1	6	9.5	Ref	
	3 < 6 year	52	36.4	9	17.3	2.0 (0.66–6.0)	0.22
	≥6 year	28	19.5	4	14.3	1.6 (0.41–6.1)	0.51
Sex	Male	67	46.9	10	14.9	Ref	
	Female	76	53.1	9	11.8	0.77 (0.29–2.0)	0.54
Sampling year	2022	98	68.5	12	12.2	Ref	
	2023	45	31.5	7	15.6	1.3 (0.48–3.6)	0.59
Frequency walking in area aquatic birds^b^	<1/week	19	13.5	0	0.0	Ref	
	≥1/week	122	86.5	18	14.8	6.9 (0.13–40.0)	0.13
Frequency swimming^b^	<1/week	13	9.2	0	0.0	Ref	
	≥1/week	128	90.8	18	14.1	4.5 (0.11–33.8)	0.37
Frequency drinking surface water	<1/week	33	23.4	3	9.1	Ref	
	≥1/week	108	76.6	15	13.9	1.6 (0.44–6.0)	0.47
Frequency wild bird contact	<1/week	76	53.9	10	13.2	Ref	
	≥1/week	65	46.1	8	12.3	0.93 (0.34–2.5)	0.88
Used as hunting dog at time of sampling	No	96	67.8	14	15.6	Ref	
	Yes	46	32.2	4	8.7	0.56 (0.17–1.8)	0.33
Bird contact in region HPAI H5 in wild birds	No	44	36.1	5	11.4	Ref	
	Yes	78	63.9	12	15.4	1.4 (0.46–4.3)	0.54
Most recent bird contact was in/near water	No	60	42.7	2	3.3	Ref	
	Yes	82	57.3	16	19.5	7.0 (1.6–31.9)	0.012
Most recent bird contact was HPAI H5 suspected bird	No	116	81.8	13	11.2	Ref	
	Yes	26	18.2	6	23.1	2.4 (0.81–7.0)	0.12
Most recent bird contact was live bird in beak	No	94	66.2	10	10.6	Ref	
	Yes	48	33.8	9	18.8	1.9 (0.73–5.2)	0.18
Most recent bird contact was eating bird faeces	No	97	67.8	14	14.4	Ref	
	Yes	46	32.2	5	10.9	0.72 (0.24–2.2)	0.56

ELISA: enzyme linked immunosorbent assay. OR: odds ratio. CI: confidence interval; Ref: reference category. For dog-bird-contact, the most recent contact within the past 6 months was used.

a Not all serum samples were accompanied by all metadata.

b Firth correction applied to adjust for categories with zero events.

In the multivariable binary logistic regression model, the dogs with “most recent bird contact in/near water” were more likely HPAI H5c ELISA seropositive (OR = 6.9; 95 % CI:1.5–31.3, *p* = 0.015) than dogs that did not.

No association was found between clinical signs (reported upon bird exposure) and HPAI H5c seropositivity in hunting-training dogs or bird-exposed dogs. None of these dogs were HPAI H5 ELISA seropositive.

#### 3.3.2 Companion dogs

In companion dogs, HPAI H5c ELISA seropositivity was more likely in dogs aged 3–6 years, compared to younger dogs (OR = 5.6) (Table 4A). Seropositivity in ELISA to HPAI H5c was more likely in dogs sampled in 2023 compared to 2022 (OR = 2.1). Seropositivity in ELISA to H1 was more likely in dogs aged 3–6 years (OR = 4.4) and aged ≥6 years (OR = 5.2) compared to younger dogs (Table 4B). In multivariable logistic regression models, age and sampling year were not significantly associated with HPAI H5c or H1 ELISA seropositivity.

**Table 4:** Analysis of factors and (**A**) HPAI H5 clade 2.3.4.4 or (**B**) human H1N1_pdm2009_ ELISA seropositivity in companion dogs.

4A		Total^a^		HPAI H5 seropositive		OR (95 % CI	*p-*value
Factors	Categories	n	%	n	%		
Age	0 < 3 year	57	16.4	1	1.8	Ref	
	3 < 6 year	55	15.9	5	9.1	5.6 (0.63–49.6)	0.12
	≥6 year	235	67.7	7	3.0	1.7 (0.20–14.3)	0.61
Sex	Female	161	46.9	5	3.1	0.8 (0.25–2.6)	0.71
	Male	182	53.1	7	3.8	Ref	
Breed	Hunting	63	18.8	2	3.2	0.78 (0.17–3.6)	0.75
	Other	272	81.2	11	4.0	Ref	
Sampling year	2022	168	48	4	2.4	0.47 (0.14–1.6)	0.21
	2023	182	52	9	4.9	Ref	


4B		Total^a^		H1 seropositive		OR (95 % CI)	*p-*value
Factors	Categories	n	%	n	%		
Age	0 < 3 yr	57	16.4	1	1.8	Ref	
	3 < 6 yr	55	15.9	4	7.8	4.4 (0.48–40.6)	0.19
	≥6 yr	235	67.7	20	9.3	5.2 (0.68–39.7)	0.11
Sex	Female	161	46.9	11	6.8	0.88 (0.39–2.0)	0.76
	Male	182	53.1	14	7.7	Ref	
Breed	Hunting	63	18.8	4	6.8	0.9 (0.30–2.8)	0.86
	Other	272	81.2	19	7.5	Ref	
Sampling year	2022	168	48.0	12	7.1	1.0 (0.44–2.3)	1
	2023	182	52.0	13	7.1	Ref	

CI: confidence interval; ELISA: enzyme linked immunosorbent assay; OR: odds ratio. Ref: reference category.

a Not all serum samples were accompanied by all metadata.

## 4 Discussion

Here, we sampled dogs for HPAI H5 and H1N1_pdm2009_ virus and/or antibodies in the Netherlands from July 2021 to October 2023. We found that hunting-training dogs were frequently and significantly more often than companion dogs, exposed to HPAI H5 virus – most likely due to increased exposure to aquatic birds.

The differences in HPAI H5 seropositivity among dog cohorts may be due to differences in lifestyle and/or duration of outdoor activities affecting direct [4,5,24] or indirect [3,24,[32], [33], [34], [35]] contact with HPAI H5-infected birds. In hunting-training dogs, this contact may have occurred at hunting-training, at hunting-trials or at hunts, or during daily walks/swims [24,36]. In contrast, in companion dogs this contact may have only occurred at daily walks/swims. Besides type of outdoor activity, prolonged outdoor time may have increased the risk of virus exposure [24,36]*.* For example; hunting-training dogs in Spain trained outdoors for 9–18 h per week [37] whereas companion dogs in the Netherlands were walked on average 2.2 h per week [38]. Due to convenience sampling and travelling of hunting-training dogs throughout the country, we refrained from drawing conclusions on the association between sampling site and HPAI H5 seropositivity. Concluding, in dogs longer duration and more diverse outdoor activities may increase the risk for HPAI H5 exposure through wild birds.

The H1 seroprevalence of hunting-training dogs (0.70 %) was low, and lower compared to companion dogs (this study 7.1 %; previous studies 1.0 % to 24.7 % [6,18,20]). As human-to-dog is the most likely H1N1_pdm2009_ IAV transmission route [19], the observed differences in seroprevalence among dog cohorts may reflect either dog associated factors or differences in human-to-dog contact. Seropositivity to H1 was more frequent in older companion dogs and may be the result of prolonged or cumulative exposure. As a consequence, the younger hunting-training dogs [mean age 3.5 years] may have been exposed less than the older companion dogs [mean age 7.3 years] [39]. Furthermore, hunting-training dogs spend more time outdoors where human-to-dog H1N1_pdm2009_ virus transmission may be less efficient than indoors [[36], [37], [38]]. None of the H1 ELISA seropositive hunting-training dogs or bird-exposed companion dogs lived on a farm, therefore we found no indication of swine-to-dog H1 influenza A virus exposure, yet can not exclude this.

Several dog sera reacted in ELISA to multiple HAs. This could be the result of cross-reacting antibodies and/or co-exposure. In three dogs, ELISA reactivity to both HPAI H5 and H1 was detected. Based on the equal or lower H1 than HPAI H5 OD ratios in these sera, and the absence of reactivity in the HAIs, we cannot distinguish co-exposure from cross-reactivity. Substantiated by the significantly higher mean rank HPAI H5c compared to LPAI H5 OD ratios of the hunting-dog sera, and the HPAI H5c- HAI positivity of three of seven samples whereas none were LPAI H5-HAI positive, we consider HPAI H5 antibodies cross-reactive to LPAI H5 [28], but cannot exclude prior LPAI H5 exposure [28].

In this study clinical signs were not associated with HPAI H5 seropositivity, and no IAV was found in necropsy dogs that displayed respiratory or neurological signs. This contrasts with reported severe or lethal cases of HPAI H5 virus infection in dogs that consumed HPAI H5-infected dead birds or contaminated raw poultry meat [2,4,5]. However, while some experimentally infected dogs displayed severe signs [12,13] most remained asymptomatic or showed only mild conjunctivitis or nasal discharge [7,[9], [10], [11]]. In addition, HPAI H5 seropositivity in absence of clinical signs has been detected in companion dogs and in hunting dogs [3,6,7,24]. It is possible that dogs are more prone to develop clinical signs after ingestion of virus, yet experimental oral HPAI H5 inoculation of dogs to confirm this hypothesis has not been conducted. The absence of detected IAV may furthermore be explained by the relatively short period of HPAI H5 virus excretion (maximum 18 days [2,[9], [10], [11], [12], [13],^19^]), or the sample type (i.e. exclusion of brain [40,41]), and/or pooling of samples [42].

Based on the owner questionnaires the factor “most recent bird contact was in/near water” was significantly associated with HPAI H5c ELISA seropositivity in hunting-training dogs. However, based on the study design a causal relationship cannot be confirmed. Due to questionnaire design, survey bias, recall bias, convenience sampling and low sample size we were limited in drawing further conclusions. Follow-up studies to explore factors driving HPAI H5 virus exposure may gather more data on high-at-risk behaviour−direct or indirect contact with aquatic birds−in larger cohorts of hunting dogs and companion dogs.

## 5 Conclusion

The high HPAI H5 seroprevalence in hunting-training dogs and high H1N1_pdm2009_ seroprevalence in companion dogs in the Netherlands indicates frequent IAV exposure. To evaluate if dogs can act as HPAI H5 virus source to other dogs or to humans, follow-up studies that explore the intensity and duration of virus excretion are needed.

We recommend dog owners to keep dogs on a leash in areas with (HPAI H5 infected-) aquatic birds. Targeted sampling for IAV or specific antibodies is advised when dogs have been exposed to sick/dead birds, or to IAV within the household, especially if dogs display respiratory signs.

## CRediT authorship contribution statement

**Mirjam B.H.M. Duijvestijn:** Writing – review & editing, Writing – original draft, Visualization, Resources, Project administration, Methodology, Investigation, Funding acquisition, Formal analysis, Data curation, Conceptualization. **Nancy N.M.P. Schuurman:** Writing – review & editing, Resources, Project administration, Methodology, Investigation, Formal analysis, Data curation. **Johannes C.M. Vernooij:** Writing – review & editing, Methodology, Investigation, Formal analysis. **Marian J. Broekhuizen:** Writing – review & editing, Project administration, Methodology, Investigation, Data curation. **Erwin de Bruin:** Writing – review & editing, Methodology, Investigation, Data curation. **Benine S. Carrière:** Writing – review & editing, Project administration, Methodology, Investigation, Formal analysis, Data curation. **Judith M.A. van den Brand:** Writing – review & editing, Supervision, Methodology, Investigation, Data curation, Conceptualization. **Jaap A. Wagenaar:** Writing – review & editing, Supervision, Resources, Methodology, Funding acquisition, Conceptualization. **Frank J.M. van Kuppeveld:** Writing – review & editing, Supervision, Resources, Methodology, Conceptualization. **Herman F. Egberink:** Writing – review & editing, Methodology, Investigation, Conceptualization. **Cornelis A.M. de Haan:** Writing – review & editing, Methodology, Investigation, Formal analysis, Conceptualization. **Josanne H. Verhagen:** Writing – review & editing, Writing – original draft, Supervision, Methodology, Investigation, Funding acquisition, Formal analysis, Conceptualization.

## Disclosure statement

No potential conflict of interest was reported by the authors.

## Funding

This work was supported by the “Jubileumfonds Diergeneeskunde”, part of the 10.13039/501100001829Utrecht University Fund, the Netherlands, under number LB237321010.

## Declaration of competing interest

The authors declare that they have no known competing financial interests or personal relationships that could have appeared to influence the work reported in this paper.

## Data Availability

Data will be made available upon reasonable request. The questionnaire and questionnaire processing is available as supplementary datafile S1.

## Appendix A Supplementary data

Supplementary material: Supplementary datafile 1: Questionnaire and questionnaire processing hunting-training dogs. Image 1
